# CircDDX17 enhances coxsackievirus B3 replication through regulating miR-1248/NOTCH receptor 2 axis

**DOI:** 10.3389/fmicb.2022.1012124

**Published:** 2022-10-13

**Authors:** Tingjun Liu, Yuhan Li, Shengjie Chen, Lulu Wang, Xiaolan Liu, Qingru Yang, Yan Wang, Xiaorong Qiao, Jing Tong, Xintao Deng, Shihe Shao, Hua Wang, Hongxing Shen

**Affiliations:** ^1^Cardiothoracic Surgery, Affiliated Hospital of Jiangsu University, Zhenjiang, China; ^2^Department of Laboratory Medicine, School of Medicine, Jiangsu University, Zhenjiang, China; ^3^Xuzhou Center for Disease Control and Prevention, Xuzhou, China; ^4^People’s Hospital of Xinghua, Jiangsu University Teaching Hospital, Xinghua, China

**Keywords:** coxsackievirus B3, circular RNAs, miRNAs, NOTCH2, METTL3

## Abstract

Coxsackievirus B3 (CVB3) was one of the most common pathogens to cause viral myocarditis. Circular RNAs as novel non-coding RNAs with a closed loop molecular structure have been confirmed to be involved in virus infectious diseases, but the function in CVB3 infection was not systematically studied. In this study, we identified that hsa_circ_0063331 (circDDX17) was drastically decreased after CVB3 infection by circRNA microarray. *In vivo* and *in vitro*, when cells or mice were infected with CVB3, the expression of circDDX17 was significantly reduced, as demonstrated by quantitative real-time PCR assays. Additionally, circDDX17 enhanced CVB3 replication by downregulating the expression of miR-1248 in HeLa and HL-1 cells, and miR-1248 regulated CVB3 replication through interacting with the gene coding for NOTCH Receptor 2 (NOTCH2), and NOTCH2 could upregulate methyltransferase-like protein 3 (METTL3). Taken together, this study suggested that circDDX17 promoted CVB3 replication and regulated NOTCH2 by targeting miR-1248 as a miRNAs sponge.

## Introduction

Coxsackievirus B3 (CVB3) is an RNA virus belonging to the *Enterovirus* genus of *Picornaviridae*. CVB3 has been identified as the most common pathogen for viral myocarditis (VM). CVB3 infections are spread worldwide, and the clinical manifestations are mainly asymptomatic or mild infections, and cold-like symptoms, but newborns and children are more likely to have severe diseases, such as pancreatitis, myocarditis, encephalitis, and type 1 diabetes (Pauschinger et al., 2004; Kühl et al., 2005).

Many factors can affect the infection of the virus, like host protein and non-coding RNAs (ncRNAs). ncRNAs include microRNAs (miRNAs), long non-coding RNA (lncRNA), and circular RNA (circRNA; Salmena et al., 2011). Circular RNAs (circRNAs) are a novel class of ncRNAs, originating from pre-mRNAs. CircRNAs are circularized by connecting a 5′ splice site with the 3′ splice site of an upstream exon or intron by a back-splicing reaction (Wang et al., 2016). Most circRNAs are composed of exons and are located in the cytoplasm; they play a significant role in regulating the translation and modification of proteins (Danan et al., 2012). In some research, it has been shown that circRNAs act as sponges for miRNAs; by forming the competing endogenous RNAs loops, circRNAs could direct binding with specific miRNAs to regulate post-transcriptional gene expression events (Memczak et al., 2013). CircBACH1 regulated hepatitis B virus by miR-200a-3p/MAP 3K2 axis (Du et al., 2022). CircSIAE inhibited CVB3 by targeting miR-331-3p (Yang et al., 2021). CircEAF2 reduced Epstein–Barr virus by miR-BART19-3p/APC/β-catenin axis (Zhao et al., 2021). These researches showed that circRNAs play an important role in infectious diseases.

The hsa_circ_0063331 (circDDX17) was formed by reverse splicing the linear transcript of exons 2–5 of the *DEAD-Box Helicase 17* (*DDX17*) gene with a length of 451 nucleotides. DDX17 was a member of the DEAD-box helicase family proteins involved in cellular RNA folding, splicing, and translation (Linder and Jankowsky, 2011). Moreover, DDX17 was involved in some virus replication, like by binding to specific stem-loop structures of viral RNA to antivirus (Moy et al., 2014). In another study, it could downregulate the expression of Epstein–Barr virus genes by YTH domain-containing proteins recruiting (Xia et al., 2021). Major studies about circDDX17 were focused on cancer, like circDDX17 as a tumor suppressor in colorectal cancer, breast cancer, and colorectal cancer (Li et al., 2018; Lin et al., 2020; Peng and Wen, 2020; Ren et al., 2020), but its function in the virus was still unclear.

N6-methyladenosine (m6A) is intimately associated with three categories of molecular compositions: “writers,” “readers,” and “easers” (Zaccara et al., 2019). Writers are m6A methyltransferases like the methyltransferase-like protein 3 (METTL3) and methyltransferase-like protein 14 (METTL14). Some research showed that METTL3 could regulate virus replication, like METTL3 inhibits Enterovirus 71 by autophagy regulation (Xiao et al., 2021), decreases syndrome coronavirus clade 2 viral load and viral gene expression in host cells (Li et al., 2021), and promotes Epstein–Barr virus infection of nasopharyngeal epithelial cells (Dai et al., 2021). NOTCH1 to 4 are transmembrane receptors that determine cell fate. The NOTCH Receptor 2 (NOTCH2) has been reported to exert distinct functions in regulating tissue homeostasis and cell fate determination (Baron, 2017; Afaloniati et al., 2020). In infectious diseases, NOTCH2 is possibly involved in regulating the Epstein–Barr virus latent/lytic status (Giunco et al., 2015), and 4.3% of hepatitis C virus-positive cells diffuse large B-cell lymphoma have NOTCH2 mutations (Arcaini et al., 2015). Previous studies have shown that NOTCH2 has some relationship with METTL3, like the Notch signaling pathway as an important downstream target of METTL3 in muscle stem cells (Liang et al., 2021), but no data showed that NOTCH2 has a direct relation with METTL3. In this study, we found silence NOTCH2 could downregulate METTL3, and overexpression NOTCH2 could upregulate METTL3. In co-immunoprecipitation analysis, METTL3 was present in the immunoprecipitated complex, and METTL3 was partially co-localized with NOTCH2 in HeLa cells.

Here, we study the effect of CVB3 infection on expression levels of circRNAs and investigate potential downstream mechanisms of their involvement in viral processes *in vivo* and *in vitro*. We examined the prevalence, regulation, and functional roles of circDDX17 in CVB3 infection. CVB3 infection could decrease the expression level of circDDX17 in cells and mics. CircDDX17 up-regulated CVB3 replication in HeLa and HL-1 cells. Furthermore, CircDDX17 regulated NOTCH2 by target miR-1,248, miR-1,248 could down-regulate NOTCH2 expression and inhibit CVB3 replication.

## Materials and methods

### Cells and virus

HeLa and HEK-293T cells were a gift from Dr. Huaiqi Jing (Chinese Center for Disease Control and Prevention). HL-1 cells were stored at the School of Medicine, Jiangsu University. Cells were cultured with Dulbecco’s modified Eagle’s medium (DMEM, Gibco, United States), supplemented with 8% fetal bovine serum (FBS, Gibco, United States), 100 U/ml penicillin, and 100 μg/ml streptomycin in 5% CO^2^ at 37°C. CVB3 (Nancy; Corsten et al., 2015) was a gift from Professor Ruizhen Chen (Department of Cardiology, Zhongshan Hospital, Shanghai, China). GFP-CVB3, expressing the green fluorescence protein (GFP; Lei et al., 2013; Shuo et al., 2014).

### Myocarditis

Coxsackievirus B3 (10^5^ PFU/mouse) was injected intraperitoneally into 3-week-old BALB/c male mice. CVB3 was diluted in 100 μl PBS for injection, and an equal volume of PBS was injected into the blank control mice (3 mice per group). This study was conducted according to the recommendations in the Guide to the Care and Use of Experimental Animals-Chinese Council on Animal Care. All protocols were approved by the Animal Care Committee of University Jiangsu (protocol number: UJS-IACUC-AP-20190307087).

### Plasmid, miRNA, siRNA, and transfection

PcicR-3.0-circDDX17 (pcircDDX17) for overexpression circDDX17, PcicR-3.0 (pcicR) for its negative control. PcDNA-3.0-NOTCH2 (pNOTCH2) for overexpression NOTCH2, used pcDNA-3.0 (pcDNA) for its negative control. miR-885 mimics (miR-885), miR-1248 mimics (miR-1248), and miR-1279 mimics (miR-1279), negative control (miR-NC); miR-1,248-inhibitor (miR-1,248-in), negative control (NC-in); siRNA-NOTCH2 (si-NOTCH2), negative control (si-NOTCH2-NC) were all synthesized by GenePharma Co., Ltd. (Suzhou, China), the sequences were listed in Table 1. Plasmid and oligonucleotide were transfected using Lipofectamine 3000^™^ (Invitrogen, United States).

**Table 1:** Sequence details.

Name	Sequence
si-circDDX17	5′ GGCCCAAUCAUUUGGAGCATT 3′
miR-1,248 mimics	5′ ACCUUCUUGUAUAAGCACUGUGCUAAA 3′
miR-885-5p mimics	5′ UCCAUUACACUACCCUGCCUCU 3′
miR-1,279 mimics	5′ UCAUAUUGCUUCUUUCU 3′
miR-1,248 inhibitor	5′ UUUAGCACAGUGCUUAUACAAGAAGGU′
si-NOTCH2-1	5′ GGCAGUGUGUGGAUAAAGUTT 3′
si-NOTCH2-2	5′ GGAGGUCUCAGUGGAUAUATT 3′
si-NOTCH2-3	5′ GUGCCAGACAGACAUGAAUTT 3′

### RNA preparation and quantitative real-time PCR

The total RNA was isolated using Trizol reagent (Invitrogen, United States). PrimeScript RT Reagent Kit (Takara, Japan) was used for reverse transcription RNA, and quantitative real-time PCR (RT-qPCR) was performed using TB Green Premix Ex TaqII (Takara, Japan). The RT-qPCR was conducted to examine the expression levels of circDDX17, mRNA levels for GAPDH, NOTCH2, and VP1, and miRNA levels for miR-885, miR-1248, miR-1279, and U6. The divergent primer was synthesized by Sangon (China), and the sequences are listed in Table 2. For RNase treatment, 2 mg of total RNA was incubated with or without 3 U/mg RNase R for 30 min at 37°C.

**Table 2:** Primer sequence details.

Name	Forward Primer (5′–3′)	Reverse Primer (5′–3′)
hsa_circDDX17	ATTTCCGTTGGCTCTTAGTG	CCTCTTGCTCCAAATGATTG
hsa-GAPDH	AGGTGAAGGTCGGAGTCAAC	GGGTGGAATCATATTGGAACA
mus_circDDX17	ATTTCCTTTGGCTCTTAGTG	CCAGCACTAGACAAATTC
mus-GAPDH	TGCCCCCATGTTTGTGATG	TGTGGTCATGAGCCCTTCC
VP1	ATTCAAGGTCCGAGTCAAC	CTGCTTGTCGTGGTGTTA
hsa-NOTCH2	CGGGGCCTACTCTGTGAAGA	ACTACGGCAAACACACAGGT
U6	CTCGCTTCGGCAGCACA	AACGCTTCACGAATTTGCGT
hsa-miR-885-5p-mimics	CTCAACTGGTGTCGTGGAGTCGGCAATTCAGTTGAGAGAGGCAG	ACACTCCAGCTGGGTCCATTACACTACC
hsa-miR-1248 mimics	CTCAACTGGTGTCGTGGAGTCGGCAATTCAGTTGAGTTTAGCAC	ACACTCCAGCTGGGACCTTCTTGTATAAGCACTG
hsa-miR-1279 mimics	CTCAACTGGTGTCGTGGAGTCGGCAATTCAGTTGAGAGAAAGAA	ACACTCCAGCTGGGTCATATTGCTT

### Cytoplasmic nucleus separation

According to the manufacturer’s instructions, nuclear plasma was extracted with a cytoplasmic nucleus extraction kit (Thermo Fisher, United States); the steps were followed as previously described (Yang et al., 2021).

### Fluorescence *in situ* hybridization (FISH)

Cy5-labeled circDDX17 probes (Jima Biotech, China) were detected in HeLa cells using a Fluorescent *in Situ* Hybridization Kit (Jima Biotech, China) following the manufacturer’s guidelines. Cell nuclei were counterstained with DAPI (Jima Biotech, China). The glass slides were analyzed and images were captured under a fluorescence microscope.

### Immunofluorescence (IF) microscopy

Cells cultured in collagen-coated chamber slides (NEST Biotechnology Co., Ltd., China) were washed and fixed with either 4% paraformaldehyde or with ice-cold methanol. Cells were permeabilized with 0.1% Triton X-100 in PBS, slides were stained with all primary antibodies (anti-METTL3, 1:200, anti-NOTCH2, 1:100), washed three times with PBS, and stained with conjugated Alexa Fluor secondary antibodies Alexa Fluor 488/594 (1200, Genetex, United States), cell nuclei were counterstained with DAPI (Jima Biotech, China). The glass slides were analyzed and images were captured under a fluorescence microscope.

### Western blot

The total proteins of the cells were extracted using the RIPA lysis buffer (Sigma, United States). Samples were subjected to 12% SDS-PAGE and transferred to polyvinylidene fluoride (PVDF) membranes (Millipore, United States). The primary antibodies against NOTCH2 (1:1,000, Sangon, China), VP1 (1:1,000, Genetex, United States), METTL3 (1:2,000, CST, United States), methyltransferase-like protein 14 (METTL14; 1:2,000, CST, United States), and GAPDH (1:20000, Genetex, United States). Membranes were blocked, incubated with secondary antibody (1:20000, Jackson, United States), and detected by electrochemiluminescence (ECL, Millipore, United States).

### Co-immunoprecipitation

For coimmunoprecipitation (co-IP) assays, 500 μg HeLa cell lysate protein was reacted with primary antibodies (10 μl) overnight at 4°C and incubated with protein A/G beads at the next day for 4 h at 4°C. Then immunoprecipitated proteins were eluted from the beads for Western blotted with indicated antibodies.

### Luciferase reporter assays

HEK-293 T cells (1 × 10^5^/well) were plated in 24-well plates. Cells were co-transfected with miR-1248 and psiCHECK-2 luciferase reporter to generate psiCHECK-2-circDDX17-wild-type (circDDX17-wt) or psiCHECK-2-circDDX17-mutant (circDDX17-mu) constructs after 48 h. Luciferase activity was determined following a dual-luciferase reporter assay detection kit (Promega, Madison, WI, United States).

### Plaque assay

Sample supernatants were collected at CVB3 7 h post-infection, serially diluted, and added onto HeLa cells in 24-well plates (1.0 × 10^5^ cells/well). After incubation for 1 h, cells were rewashed with PBS three times, overlaid with 0.75% soft agar medium, and incubated for 3 days. Cells were fixed with glacial acetic acid for 30 min and stained with 1% crystal violet. All the assays were conducted at least in triplicate.

### Statistical analysis

Statistical analysis was performed using Graph Pad Prism 7 (GraphPad Software Inc., San Diego, CA, United States). The group difference was evaluated by Student’s *t*-test, error bars represent mean ± SD and the difference was statistically significant when ^*^p < 0.05, ^**^p < 0.01, or ^***^p < 0.001.

## Results

### Result 1. Characterization of the existence and subcellular distribution of circDDX17 in HeLa cells

**Figure 1:**
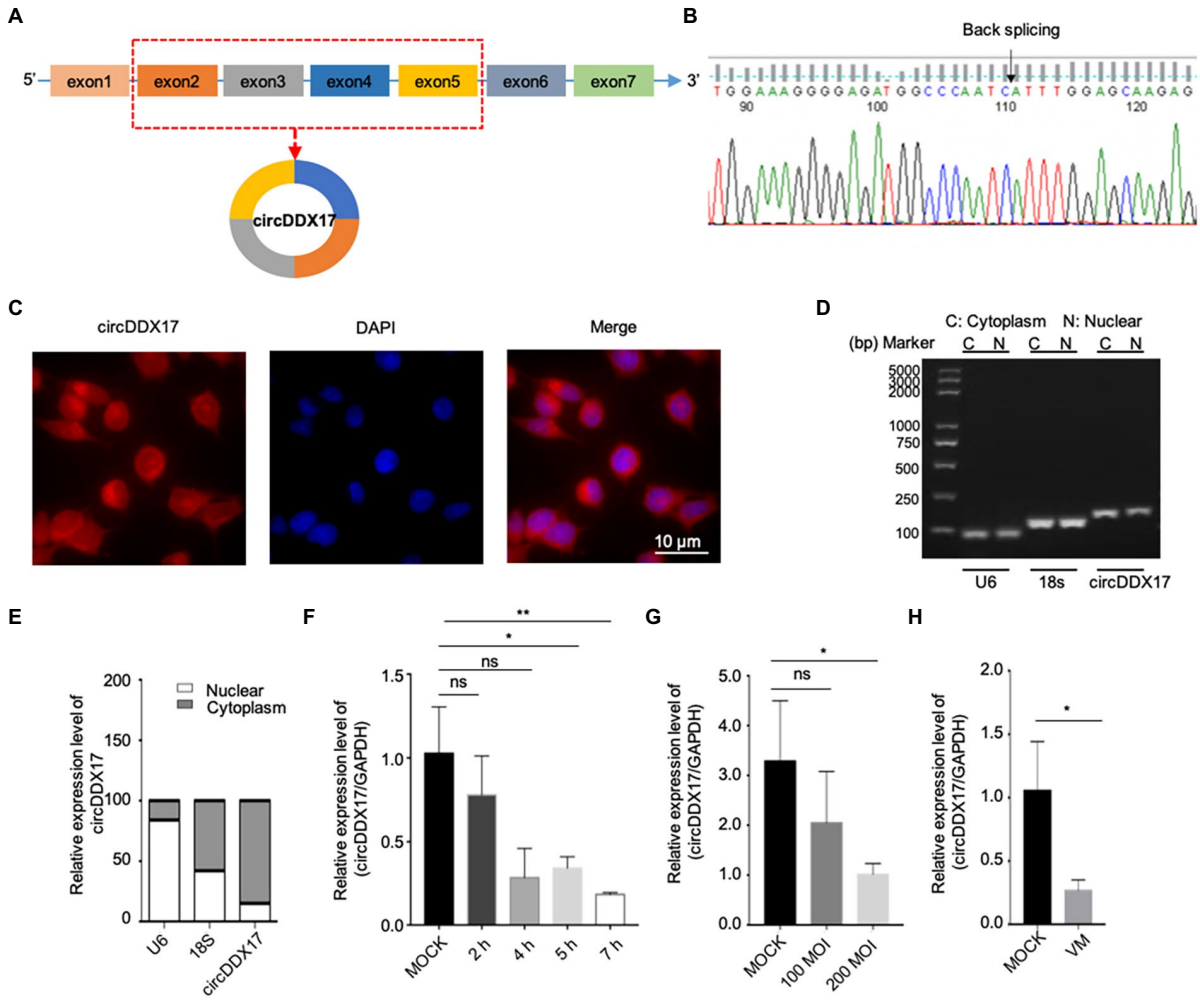
Characterization and subcellular distribution of circDDX17 in HeLa cells. **(A,B)**. Sanger sequencing confirmation of the head-to-tail splicing of circDDX17. **(C–E)** The sub-cellular distribution of circDDX17 was mostly present in the cytoplasm by the nuclear mass. **(F)** RT-qPCR analysis of circDDX17 expression in HeLa cells 7 h after CVB3 infection. **(G)** RT-qPCR analysis of circDDX17 in CVB3-infected HL-1 cells (100 MOI and 200 MOI) for 24 h. **(H)** RT-qPCR analysis of circDDX17 in viral myocarditis, MOCK mice were as control. *p < 0.05, **p < 0.01.

The Circbank database showed that circDDX17 is formed by reverse splicing of the linear transcript of exons 2–5 of the *DDX17* gene with a length of 451 nucleotides. To further characterize circDDX17, Sanger sequencing was performed to confirm head-to-tail splicing (Figures 1A,B). FISH analysis (Figure 1C) and nuclear separation assay (Figures 1D,E) was conducted to determine the subcellular localization of circDDX17 in HeLa cell lines.

To investigate the roles of circDDX17 in CVB3 infection, HeLa and HL-1 cells were infected with CVB3, and circDDX17 levels were examined. In HeLa cells, at the early stages of infection (0–2 h), no significant change in circDDX17 expression was detected between CVB3-infected and mock control cells. At 4–7 h post-infection, the circDDX17 expression was significantly decreased compared to MOCK cells (Figure 1F). The expression level of circDDX17 in HL-1 cells infected with 200 MOI CVB3 was lower than 100 MOI CVB3 at 24 h post-infection (Figure 1G). In VM mice, the circDDX17 expression level was lower than in MOCK mice (Figure 1H).

### Results 2. CVB3 infection reduced circDDX17 expression level, and circDDX17 promotes CVB3 replication

**Figure 2:**
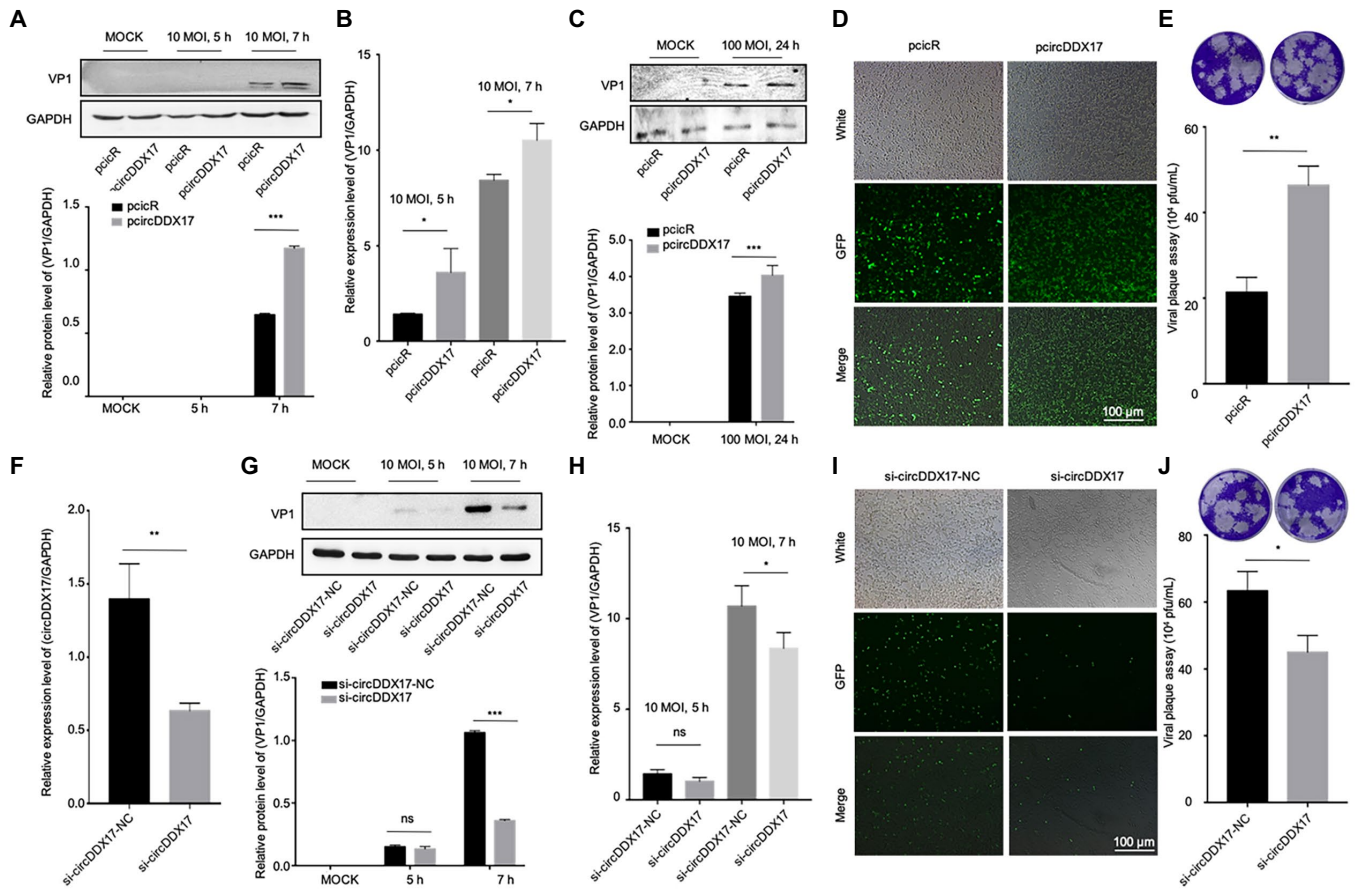
CircDDX17 promotes CVB3 replication. **(A,B)** Western blot and RT-qPCR of VP1 expression levels in CVB3-infected HeLa cells overexpressing circDDX17 (Western blot and analyzed using ImageJ software). **(C)** Western blot to analyze the expression of VP1 after HL-1 overexpression circDDX17. **(D)** HEK-293T cells co-transfected pcircDDX17 and GFP-CVB3, observed GFP-positive cell number after 48 h. **(E)** Viral plaque assay. Viral titer was determined by plaque assay using the supernatants collected at 7 h (*n* = 3). **(F)** RT-qPCR to analyze the circDDX17 expression after si-circDDX17 transfected HeLa cells. **(G,H)** Western blot and RT-qPCR to detect VP1 expression in HeLa cells after transfected with the si-circDDX17 and infected CVB3 (10 MOI). **(I)** HEK-293T co-transfected with si-circDDX17 and GFP-CVB3, observed GFP-positive cell number after 48 h. **(J)** Viral titers were determined by plaque assay using the supernatants (*n* = 3). *p < 0.05, **p < 0.01, ***p < 0.001.

HeLa cells (Figures 2A,B) and HL-1 cells (Figure 2C) overexpressed circDDX17 were infected with CVB3 to analyze the expression of VP1. The results showed that circDDX17 increased the expression of VP1 after CVB3 infection. HEK-293 T cells co-transfected with pcicR-3.0-circDDX17 (pcircDDX17) and GFP-CVB3, cells overexpressed circDDX17 GFP-positive cell number were observed more than cells co-transfected with pcicR-3.0 (pcicR) and GFP-CVB3 (Figure 2D). To gain further insight into the function of cricDDX17 on CVB3 replication, a viral plaque assay was adopted. The results showed that cricDDX17 overexpression increased viral release compared to the control (Figure 2E).

In contrast, circDDX17 silencing decreased the expression of VP1 at CVB3 after CVB3 infection (Figures 2F–H). HEK-293T cells co-transfected with siRNA-circDDX17 (si-circDDX17) and GFP-CVB3 showed lower GFP-positive cells than the cells transfected siRNA-circDDX17-NC (si-circDDX17-NC) and GFP-CVB3 (Figure 2I), and circDDX17 silencing could reduce viral release (Figure 2J).

### Results 3. CircDDX17 promotes CVB3 replication *via* the miR-1248/NOTCH2 axis

**Figure 3:**
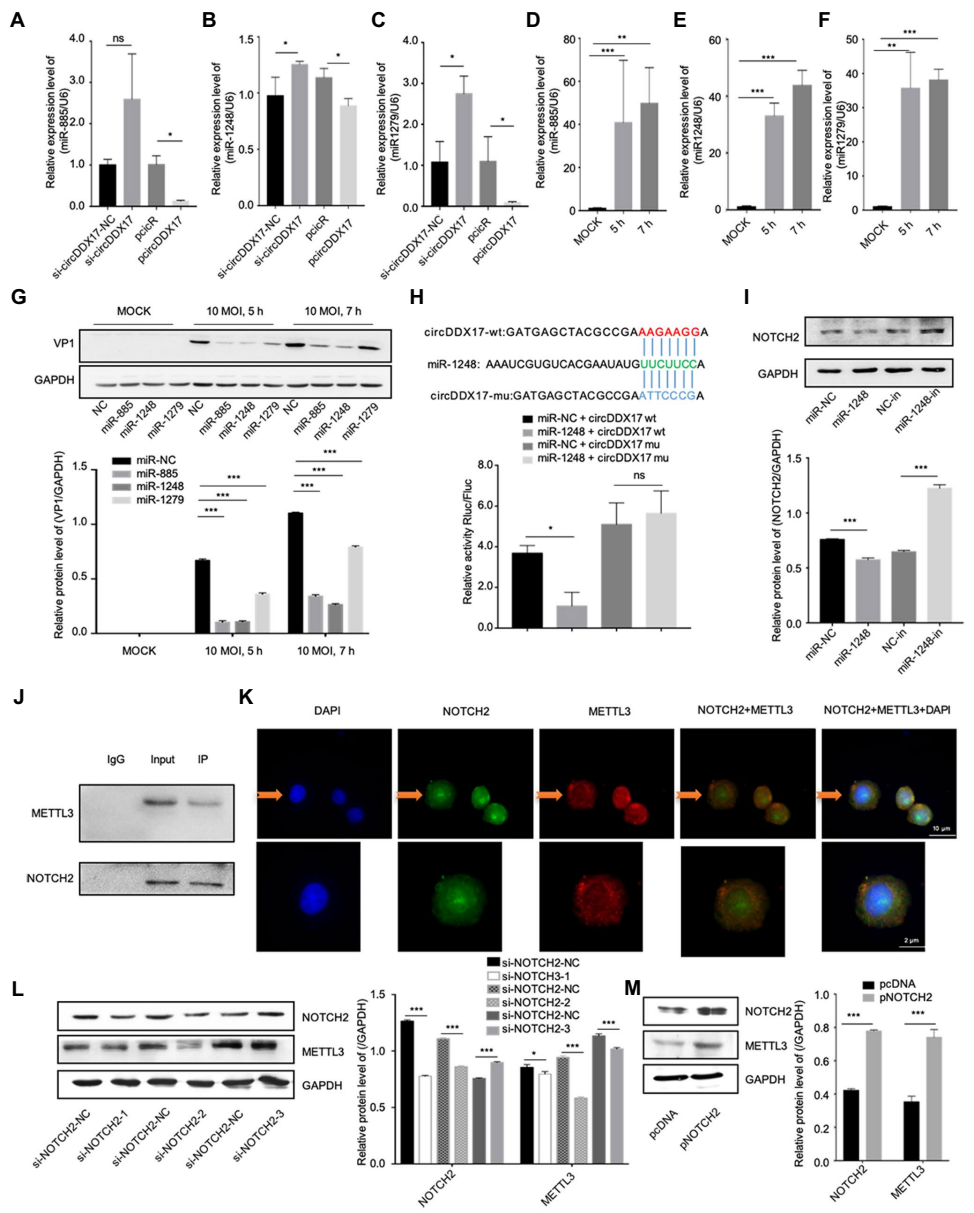
CircDDX17 promotes CVB3 replication by targeting miR-1248, and miR-1248 targeting NOTCH2. **(A–C)** Relative expression levels of miR-885, miR-1248, and miR-1279 in HeLa cells were determined by RT-qPCR after cells overexpression or silence the circDDX17. **(D–F)** RT-qPCR to detected expression of miR-885, miR-1248, and miR-1279 in CVB3 (10 MOI) infected HeLa cells. **(G)** HeLa cells transfected with miR-885, miR-1248, and miR-1279, infected the CVB3 (10 MOI) for 7 h. Western blot analysis of the VP1 expression. **(H)** The putative miR-1248 binding site in circDDX17 (circDDX17-wt) and the designated mutant sequence (circDDX17-mu) are illustrated. Validation of circDDX17 targeting on miR-1248 luciferase assay. **(I)** HeLa cells transfected miR-1248 mimics or miR-1248-in, indicated NOTCH2 detected by Western blot. **(J)** Immunoprecipitation and immunoblot analyses were performed with the indicated antibodies. **(K)** HeLa cells stained with anti-NOTCH2 (green) and anti-METTL3 (red) antibody and were analyzed by confocal microscope. Nuclei were labeled with DAPI (blue). Micrographs with × 40 magnification (scale bar of 10 μm) are shown. **(L)** HeLa cells transfected si-NOTCH2, indicated signals were detected by Western blot. **(M)** NOTCH2 overexpression in HeLa cells. Indicated signals were detected by Western blot. *p < 0.05, **p < 0.01, ***p < 0.001.

CircRNAs have been shown to act as a miRNA sponge to regulate gene expression (Guo et al., 2020); therefore, the potential miRNAs associated with circDDX17 were investigated. First, through bioinformatics analysis, three miRNAs (miR-885, miR-1248, and miR-1279) were identified from the overlap of three databases (circBank, miRanda, and CircInteractome) as possible targets for circDDX17. RT-qPCR of HeLa cells transfected with pcircDDX17 or si-circDDX17 showed that circDDX17 downregulates the miRNAs expression (Figures 3A–C). To analyze the role of CVB3 on miRNA expression, total RNA from CVB3 infected cells was collected for RT-qPCR. miR-885, miR-1248, and miR-1279 expression increase with virus infection (Figures 3D–F). To investigate the role of miRNAs in CVB3 infection, HeLa cells were transfected with miRNAs mimics, and the results showed that miR-885, miR-1248, and miR-1279 could inhibit the replication of CVB3, among which miR-1248 had the most obvious effect (Figure 3G), so we chose miR-1248 as the object of study. The dual-luciferase reporter assay confirmed the direct interaction between circDDX17and miR-1248. The circDDX17-wild-type (circDDX17-wt) and circDDX17-mutant (circDDX17-mu) full-length sequences without miR-1248 binding sites were cloned into the luciferase vector. Subsequently, luciferase reporter assays confirmed that miR-1248 mimics markedly reduced the luciferase activity of circDDX17-wt but not that of circDDX17-mu compared to the miR-NC group (Figure 3H). To investigate the signal pathways contributing to the miR-1,248 effect on CVB3 replication, we sought to identify its target genes. Bioinformatic analyses using TargetScan, miRDB, and miRWalk programs showed that NOTCH2 is one of the predicted targets. HeLa cells were transfected with miR-1248 mimics (miR-1248) or miR-1248-inhibitor (miR-1248-in), and the results showed that miR-1248 has a negative regulatory role on NOTCH2 expression (Figure 3I).

In previous research, NOTCH2 has a relationship between METTL3 and DNA-methylation (Terragni et al., 2014), we predicted METTL3 as NOTCH2 interaction protein by String,^1^ therefore, HeLa cells were transfected with specific si-NOTCH2 or pNOTCH2 to silence or overexpressed NOTCH2. For further verification, we performed coimmunoprecipitation experiments to study the relationship between NOTCH2 and METTL3 in HeLa cells (Figure 3J), the result showed that the METTL3 was present in the immunoprecipitated complex. As shown in Figure 3K, METTL3 and NOTCH2 were distributed in the nucleus although a small fraction of these proteins were also found in the cytoplasm, and METTL3 was partially co-localized with NOTCH2. The Western blot results show that NOTCH2 regulates METTL3 expression, METTL3 increases with the increasing expression level of NOTCH2 and decreases with NOTCH2 decreasing expression level (Figures 3L,M).

### Results 4. CircDDX17 promotes CVB3 replication, and rise DNA-methylation-associated protein METTL3 and METTL14 expression

**Figure 4:**
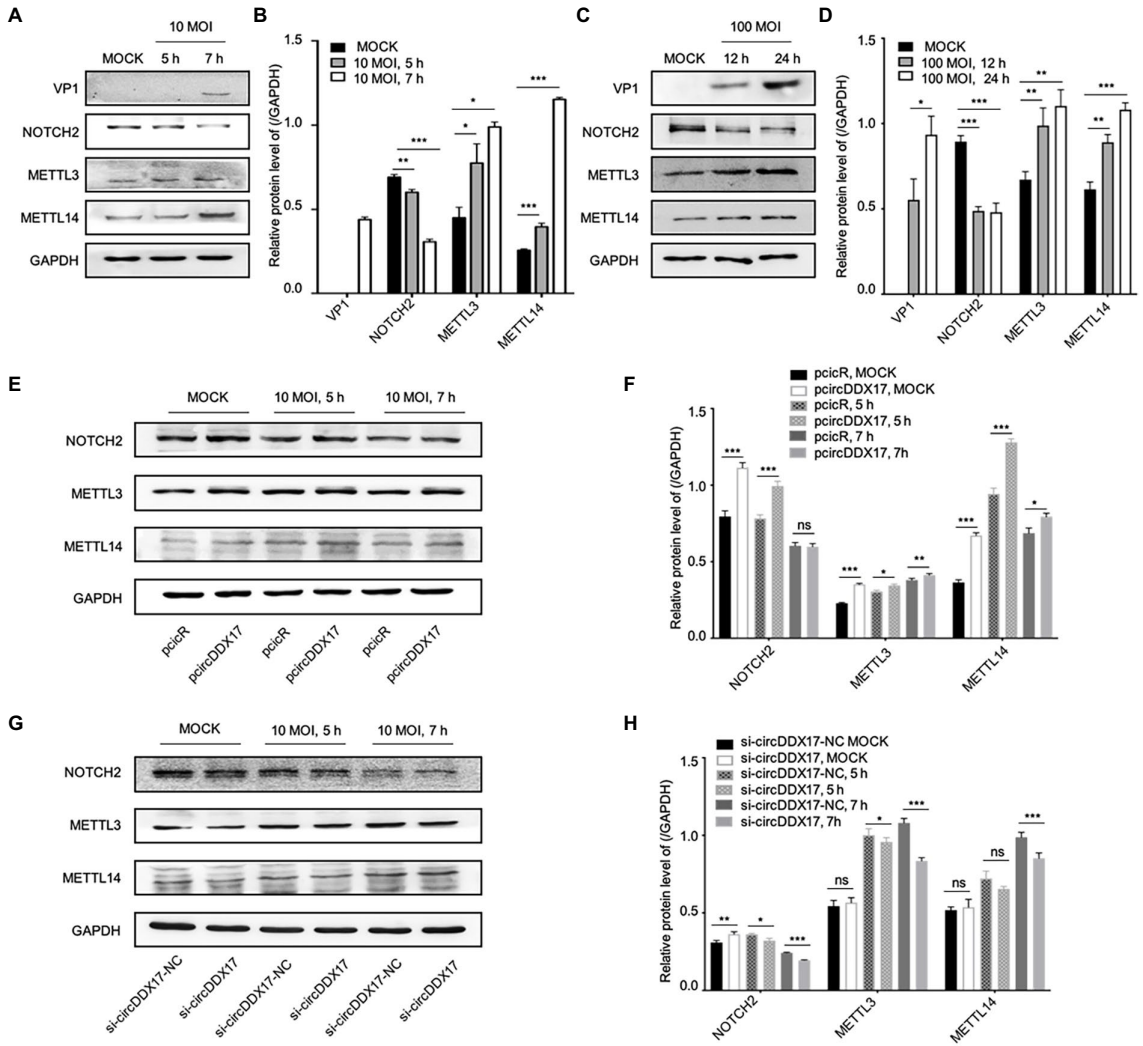
CircDDX17 regulates NOTCH2 expression and influences methylation-related pathways after CVB3 infection. **(A,B)** HeLa cells infected CVB3 (10 MOI) for 7 h, signals were detected by Western blot. **(C,D)** HL-1 cells infected CVB3 (100 MOI) for 24 h, signals were detected by Western blot. **(E,F)** HeLa cells overexpression circDDX17 by pcircDDX17, cells infected CVB3 (10 MOI) for 7 h, indicated signals were detected by Western blot. **(G,H)** HeLa cells silenced circDDX17 by si-circDDX17, cells infected CVB3 (10 MOI) for 7 h, indicated signals were detected by Western blot. *p < 0.05, **p < 0.01, ***p < 0.001.

To elucidate the mechanism of CVB3 upregulation of host circDDX17, the expressions of DNA-methylation-associated proteins METTL3, and METTL14 were examined. With CVB3 infection, NOTCH2 declined gradually, and METTL3 and METTL14 were elevated (Figures 4A–D). To understand the roles of NOTCH2 in circDDX17-mediated DNA-methylation, we examined the downstream effector gene expression after CVB3 infection in HeLa cells overexpressing or silencing circDDX17. Western blot analysis showed that overexpression of circDDX17 upregulated the expression level of NOTCH2, METTL3, and METTL14 (Figures 4E,F). Conversely, circDDX17 silencing decreases NOTCH2, METTL3, and METTL14 expression levels (Figures 4G,H).

### Results 5. CircDDX17 promotes CVB3 replication by targeting miR-1248.

**Figure 5:**
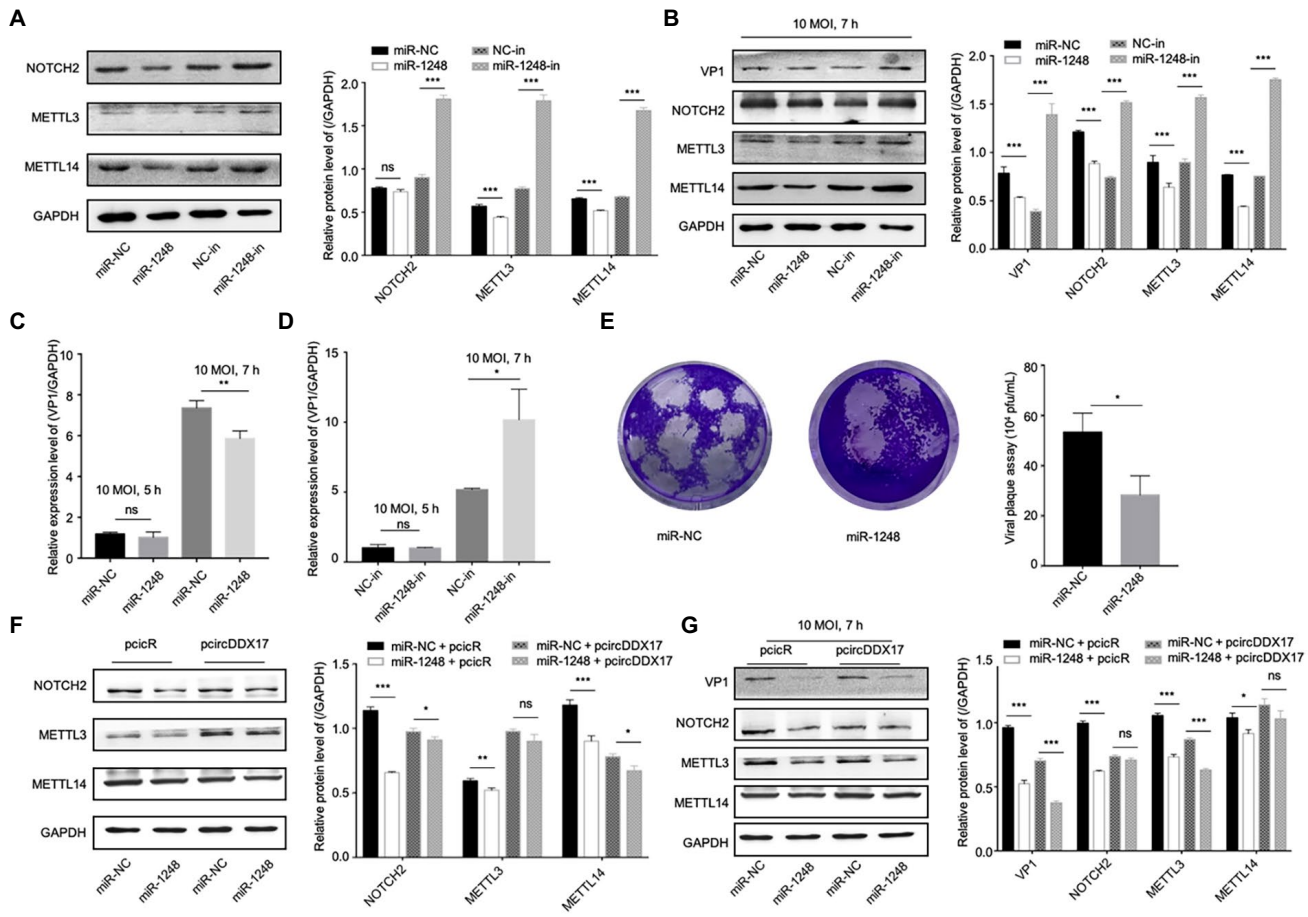
CircDDX17 regulates NOTCH2 expression by targeting miR-1248, influence the methylation-related pathway after CVB3 infection. **(A)** HeLa cells transfected miR-1248 mimics or miR-1248-inhibitor, indicated signals were detected by Western blot. **(B)** HeLa cells transfected miR-1248 mimics or miR-1248-inhibitor, and infected CVB3 (10 MOI) for 7 h indicated signals were detected by Western blot. **(C)** HeLa cells transfected the miR-1248, infected CVB3 (10 MOI) for 7 h, RT-qPCR to detect the VP1 expression. **(D)** HeLa cells silenced miR-1248 and infected CVB3 (10 MOI) for 7 h, RT-qPCR to detect the VP1 expression. **(E)** Viral titers were determined by plaque assay using the supernatants of HeLa cells overexpression miR-1,248 (*n* = 3). **(F)** HeLa cells co-transfected miR-1248 and pcircDDX17, indicating signals were detected by Western blot. **(G)** HeLa cells co-transfected miR-1248 and pcircDDX17, cells infected CVB3 (10 MOI) for 7 h, indicated signals were detected by Western blot. *p < 0.05, **p < 0.01, ***p < 0.001.

To study the effect of miR-1,248 in NOTCH2, METTL3, and METTL14 expression, HeLa cells were transfected with miRNA mimics or miRNA inhibitors, Western blot showed that miR-1248 played a negative role on NOTCH2 expression, and so did METTL3 and METTL14 expression levels (Figure 5A). Then cells infected with CVB3, miR-1248 decreased VP1 expression while inhibiting miR-1248 increased VP1 expression, and miR-1248 down-regulate the NOTCH2, METTL3, and METTL14 expression (Figure 5B).

HeLa cells overexpressing miR-1248 reduced CVB3 replication and cells silencing miR-1248 increased CVB3 replication (Figures 5C,D). At the same time, miR-1248 decreased CVB3 released as detected by viral plaque assay (Figure 5E).

To further confirm that miR-1248 downregulation by circDDX17 benefits CVB3 replication, we overexpressed miR-1248 in the presence of circDDX17 by co-transfection, and cells without CVB3 (Figure 5F) or infected CVB3 (Figure 5G). Western blot showed that compared with miR-NC + PcicR, overexpression of miR-1248 inhibited VP1, NOTCH2, METTL3, and METTL14 expression.

### Results 6. MiR-1248 inhibits CVB3 replication by targeting NOTCH2

**Figure 6:**
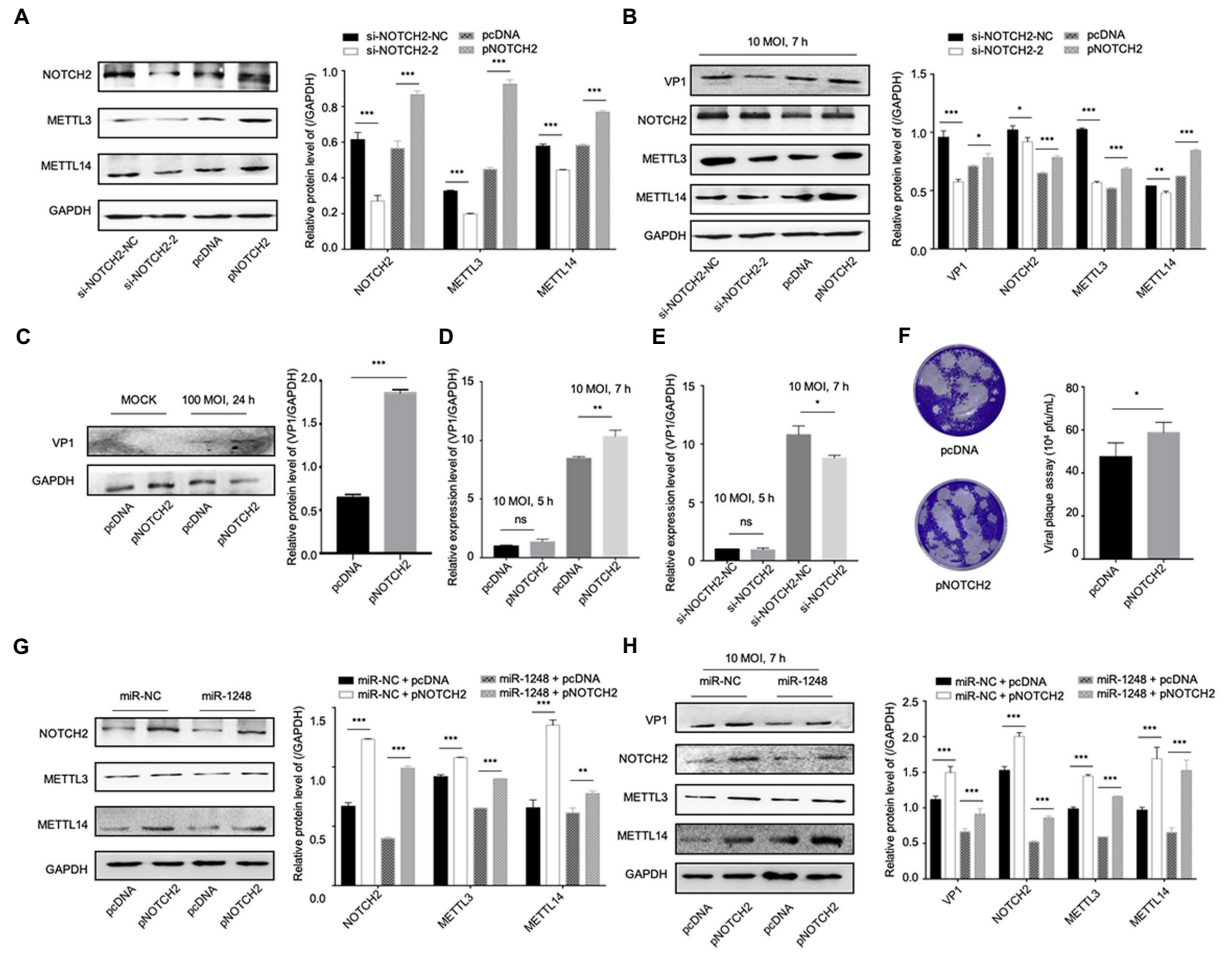
MiR-1248 regulates CVB3 replication by targeting NOTCH2 through methylation-related pathways. **(A)** HeLa cells transfected with pNOTCH2 or pcDNA, detected by Western blot. **(B)** HeLa cells transfected with pNOTCH2 or pcDNA and infected CVB3 (10 MOI) for 7 h, detected by Western blot. **(C)** HL-1 cells transfected the pNOTCH2, infected CVB3 (100 MOI) for 24 h, and Western blot analyzed the VP1. **(D,E)** HeLa cells transfected pNOTCH2 or si-NOTCH2-2, infected CVB3 (10 MOI) for 7 h, and RT-qPCR to detect the VP1 expression. **(F)** Viral titers were determined by plaque assay using the supernatants (*n* = 3). **(G)** HeLa cells co-transfected miR-1248 and pNOTCH2, Western blot to analyze the signals. **(H)** HeLa cells co-transfected miR-1248 and pNOTCH2, cells infected CVB3 (10 MOI) for 7 h, indicated signals were detected by Western blot. *p < 0.05, **p < 0.01, ***p < 0.001.

To understand the roles of NOTCH2 in circDDX17 and miR-1248-mediated methylation-related pathways, we first confirmed NOTCH2 function on methylation-related proteins. Western blot data showed that independent of CVB3 infection, METTL3, and METTL14 expression was reduced by si-NOTCH2 transfection and induced in pNOTCH2 transfection (Figure 6A). In HeLa cells, VP1 expression was decreased by si-NOTCH2 and increased by pNOTCH2 expression (Figure 6B). Furthermore, in HL-1 cells, overexpression of NOTCH2 increased VP1 expression compared to the negative control (Figure 6C). NOTCH2 could significantly increase VP1 expression level, NOTCH2 deficiency repressed VP1 expression (Figures 6D,E), and overexpression NOTCH2 increased viral release (Figure 6F).

To further confirm miR-1248 regulated CVB3 replication by targeting NOTCH2, HeLa cells overexpression miR-1248 and NOTCH2 by co-transfection (Figures 6G,H). Without CVB3 infection, cells transfection miR-NC + pNOTCH2 the METTL3 and METTL14 expression levels were higher than cells transfection miR-1248 + pNOTCH2 (Figure 6G). Seven hours post CVB3 infection, miR-NC + pNOTCH2 increased the production of VP1, METTL3, and METTL14 compared with miR-NC + pcDNA. Co-transfection of pNOTCH2 and miR-1248 reduced the production of VP1, METTL3, and METTL14 compared to miR-NC + pNOTCH2 (Figure 6H).

## Discussion

Coxsackievirus B3 is the commonest pathogen for acute and chronic myocarditis (Pauschinger et al., 2004; Esfandiarei and McManus, 2008). After CVB3 entry into the cardiomyocytes, the virus replicates and induces cell damage, triggering the host immune responses. If the virus cannot be eliminated, myocarditis can become chronic, triggering extensive myocardial fibrosis and the development of dilated cardiomyopathy (Kawai, 1999; Garmaroudi et al., 2015). In our previous study, miR-324-3p inhibits CVB3 replication by targeting the tripartite motif 27 (Liu et al., 2021), but there are fewer studies on circRNA regulation of CVB3 replication. CircRNA can play a role as a miRNA sponge to influence miRNA expression and regulate gene function.

This study identified that circDDX17 was a novel regulator of CVB3 replication. MiR-1248, a target miRNA of circDDX17, played a negative role in replicating CVB3 in host cells. Moreover, NOTCH2 was the miR-1248 target gene. NOTCH2 has been involved in cardiac fibrosis, regulating heart development and multiple antiviral immune responses. In addition, NOTCH2 mutations resulted in multiple cardiac diseases and vascular anomalies (Pinkert et al., 2019). Interestingly, NOTCH2 was distributed in m6A modification proteins. m6A was a conserved internal modification found in almost all eukaryotic nuclear RNAs (Jia et al., 2013) and the viral RNA. m6A was dynamic methylation involved in RNA metabolism, splicing, and decay (Roundtree et al., 2017; Zhao et al., 2017). METTL3 could modulate the NOTCH signaling pathway (Wang et al., 2020). In our study, there was a positive correlation between NOTCH2 and METTL3. By the analysis of IP, METTL3 was present in the immunoprecipitated complex, and METTL3 was partially co-localized with NOTCH2, those results showed that METTL3 and NOTCH2 have interaction in cells. METTL3 could negatively regulate type I interferon response by dictating the fast turnover of interferon mRNAs for antivirus (Winkler et al., 2019), and METTL3 boosted Enterovirus 71 replication (Hao et al., 2019), which might explain how NOTCH2 regulates viral replication. In another way, m6A modification was dynamically and reversibly regulated by the “writers” complex (METTL3 and METTL14; Liu et al., 2014). Our study analyzed METTL3 and METTL14 as targets indicating m6A modification changes and function in cells overexpressing or silencing circDDX17 infected with CVB3. The results showed that CVB3 infection could increase the METTL3 and METTL14 expression. METTL14 played an important role in the transcription of IFNs and inflammatory cytokines, and regulates antivirus innate immunology response (Xu et al., 2021). However, in this research, we have not studied the effect of METTL14 on the replication of CVB3.

In conclusion, this study reported that circDDX17 promotes CVB3 replication by regulating miR-1248 and NOTCH2/METTL3. These findings enriched our understanding of the functional roles of circRNA in viral replication and provided novel insights into the development of therapeutic strategies.

## Data availability statement

The datasets presented in this study can be found in online repositories. The names of the repository/repositories and accession number(s) can be found in the article/Supplementary material.

## Ethics statement

The animal study was reviewed and approved by Animal Care Committee of University Jiangsu (protocol number: UJS-IACUC-AP-20190307087).

## Author contributions

HS and HW conceived and designed the experiments. TL, YL, XL, QY, YW, and XQ performed the experiments. HS, HW, and SS analyzed the data. TL, HS, HW, JT, XD, and SC contributed reagents, materials, and analysis tools. TL, HS, and HW wrote the paper. All authors contributed to the article and approved the submitted version.

## Funding

The research was supported by National Natural Science Foundation of China, grant no. 81971945 (https://isisn.nsfc.gov.cn/egrantweb/).

## Conflict of interest

The authors declare that the research was conducted in the absence of any commercial or financial relationships that could be construed as a potential conflict of interest.

## Publisher’s note

All claims expressed in this article are solely those of the authors and do not necessarily represent those of their affiliated organizations, or those of the publisher, the editors and the reviewers. Any product that may be evaluated in this article, or claim that may be made by its manufacturer, is not guaranteed or endorsed by the publisher.

## Footnotes

^1^ https://cn.string-db.org/
