# Supplementary material for: CircDDX17 enhances coxsackievirus B3 replication through regulating miR-1248/NOTCH receptor 2 axis
Source: Front Microbiol. 2022 Oct 13;13:1012124. doi: 10.3389/fmicb.2022.1012124 (PMC9627658; doi:10.3389/fmicb.2022.1012124)
Supplement: Supplementary file 1 [file Data_Sheet_1.DOCX]

The Raw date

CircDDX17 enhances coxsackievirus B3 replication through regulating miR-1248/NOTCH Receptor 2 axis

**Running** **title**: CircDDX17 improve CVB3 replication by targeting miR-1248/NOCTH2

**Name (s) of author (s)**： Tingjun Liu^2†^, Yuhan Li^2†^, Lulu Wang^2^, Xiaolan Liu, Qingru Yang^2^, Yan Wang^2^, Xiaorong Qiao^2^, Jing Tong^3^, Xintao Deng^4^, Shengjie Chen^1^, Shihe Shao^2^, Hua Wang^2^*, Hongxing Shen^1,2^*

*https://www.jianguoyun.com/c/sd/159a1a3/5c842e32a916f76b#from=https%3A%2F%2Fwww.jianguoyun.com%2Fc%2Fsd%2F159a1a3%2F5c842e32a916f76b*
